# Phosphomolybdic acid directed phosphomolybdic acid directed assembly of MoO_*x*_ into all-inorganic dendrimer-like flexible superstructures

**DOI:** 10.1039/d6ra03230c

**Published:** 2026-08-14

**Authors:** Fozia Nazir, Syeda Sundas Musawar, Bilal Akram, Ashfaq Ahmed Khan

**Affiliations:** a Department of Chemistry, Women University of Azad Jammu and Kashmir Bagh 12500 Pakistan bai-l16@mails.tsinghua.org.cn

## Abstract

A one-step hydrothermal strategy is reported for the synthesis of dendrimer-like all-inorganic phosphomolybdic acid molybdenum oxide (PMO) superstructures assembled from phosphomolybdic acid clusters and MoO_*x*_ nuclei. Hierarchical organization of primary clusters into bead-like intermediates and subsequently into three-dimensional branched networks was confirmed by TEM, SEM, and STEM-EDS analyses, revealing uniform morphology and homogeneous elemental distribution. Control experiments highlight the critical role of phosphomolybdic acid in directing anisotropic assembly. The resulting material exhibits a distinct and tunable UV-vis response toward Pb^2+^ ions, demonstrating its potential as a colorimetric sensing platform. This work introduces a modular biomimetic route to complex inorganic architectures, opening new avenues for the design of advanced functional materials.

## Abstract 2

A one-step hydrothermal strategy is reported for the synthesis of dendrimer-like all-inorganic phosphomolybdic acid molybdenum oxide (PMO) superstructures assembled from phosphomolybdic acid clusters and MoO_*x*_ nuclei.

## Graphical abstract

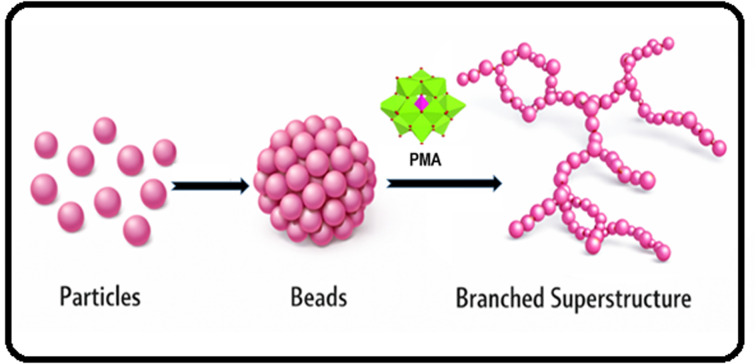

## Introduction

Superstructures, the architectural masterpieces of chemistry, are hierarchically organized assemblies in which atoms, molecules, or nanoscale building blocks combine to produce emergent properties that surpass those of the individual components.^1,2^ These highly ordered frameworks arise from the programmed arrangement of matter across multiple length scales, giving rise to dynamic behaviors, cooperative functions, and material properties that cannot be achieved at the molecular level alone.^1,3^ Superstructures encompass diverse classes including micelles, vesicles, supramolecular polymers, gels, nanotubes, nanoribbons, coordination cages, metal–organic frameworks (MOFs), covalent organic frameworks (COFs), core–shell or multilayered particles, and dendrimer-like or hyperbranched architectures.^4,5^ Their assembly is typically governed by directional, reversible forces: hydrogen bonding, π–π stacking, metal–ligand interactions, host–guest inclusion, and electrostatics, which provide stimuli-responsiveness, self-healing ability, and dynamic adaptability.^3,6^ The resulting structures exhibit emergent functionalities such as tunable porosity, high surface area, cooperative catalysis, signal amplification, and enhanced mechanical/thermal stability, enabling applications in catalysis, separation, sensing, energy conversion, and drug delivery.^4,6^

Within this broad landscape, dendrimer-like superstructures form a distinctive subclass. These include monodisperse dendrimers as well as dendrimer-inspired systems such as dendrimer-like porous nanoparticles, star polymers, dendronized polymers, and hyperbranched materials that replicate dendritic topologies and multivalency.^7,8^ Such structures can be produced *via* classical divergent/convergent dendrimer synthesis or *via* bottom-up and templated strategies that generate dendritic surface features or graded pore networks on inorganic cores.^9,10^ Dendrimer-based and dendrimer-inspired assemblies offer high surface functionality, internal cavities, controllable multivalency, and well-defined nanoarchitectures, enabling their use in encapsulation, targeted delivery, heterogeneous catalysis, plasmonic/SERS-active hybrids, and nanoscale templating.^7,9,11^

Recent advances in nanomaterial synthesis have produced dendrimer-like, branched (“tree-like”) nanoarchitectures across a variety of inorganic systems.^12^ In electrodeposition methods, metals such as Zn or Cu can be grown into dendritic nanostructures by tuning electrochemical conditions, yielding highly branched, high-surface-area morphologies.^13^ Hierarchical metal-oxide materials prepared *via* sol–gel self-assembly also enable formation of dendritic networks with large mesopores and macropores, mimicking the branching pattern of dendrimers.^14^ Template directed growth further facilitates dendritic architectures; for example, Ni–Co layered double hydroxide nanosheets grown radially around reduced graphene oxide produce 3D “cell-like” dendritic structures.^15^ Similarly, combining a dendritic copper base with Co–Ni LDH nanosheets yields a composite featuring a branched metal core and a layered dendritic shell.^16^ These strategies collectively provide precise control over branching, surface area, and hierarchical geometry, expanding the range of dendrimer-inspired nanomaterials available for catalysis, energy storage, and sensing.

Researchers have developed a wide array of dendrimer-like architectures hyperbranched or star-branched polymeric and nanoparticulate systems that replicate many of the advantageous features of true dendrimers (*e.g.*, high terminal functional group density, compact globular shape, tunable solubility, and multifunctionality) while avoiding the synthetic complexity and cost associated with perfectly monodisperse dendrimers. One notable example is hyperbranched polyglycerols (HPGs), which have been widely explored for drug delivery due to their facile one-pot synthesis, biocompatibility, and multivalent surface.^17–19^ Similarly, lysine-based dendrigrafts (dendritic polypeptides) have been applied in biomedical scaffolds; for example, star-shaped poly-l-lysine architectures initiated from dendritic cores offer degradability and efficient gene-delivery capability.^20,21^ Another strategy employs multi generation star-branched polymers such as PMA or polystyrene prepared by living anionic polymerization with interactive chain joining steps to yield dendrimer-mimetic stars.^22^ In the inorganic–organic realm, hierarchical mesoporous silica nanoparticles with dendritic (radial) pore architectures have been synthesized to provide dendrimer-like high surface area and multivalent cargo loading.^23^ Finally, hyperbranched polyurethane triazoles produced in a single-pot azide–alkyne “click” polymerization exhibit a controllable degree of branching and tunable hydrodynamic radius, offering a scalable route to multifunctional dendritic-like networks.^24,25^ Despite significant progress in the design of dendritic and dendrimer-inspired nanostructures, most reported strategies still rely on multistep organic syntheses or templating methods that afford only limited control over branching and composition.^26,27^

In contrast, the construction of dendrimer-like architectures exclusively from discrete, compositionally unique inorganic units remains largely unexplored. Here, we introduce a new synthetic strategy that integrates two structurally rigid, building blocks phosphomolybdic acid (PMA) and MoO_*x*_ to produce a well-defined, branched nanoarchitecture resembling a dendrimer. PMA serves not only as a robust polyoxometalate node but also imparts intrinsic Brønsted acidity, redox activity, and oxidative stability to the resulting framework, enabling multifunctionality without post-synthetic modification,.^17,28–30^ The resulting material phosphomolybdic acid molybdenum oxide (PMO) superstructure is not a dendrimer in the classical macromolecular sense, but rather a dendrimer-like inorganic superstructure synthesized through a single-step, modular method that circumvents the synthetic limitations typically associated with dendrimer construction. To the best of our knowledge, this represents a rare example of a dendrimer-inspired architecture produced entirely from inorganic precursors, establishing a new strategy for fabricating hierarchically branched nanomaterials with tunable topology and potential utility in catalysis, redox chemistry, and molecular host–guest applications.^30–34^

## Experimental

### Materials and method

Phosphomolybdic acid (PMA, H_3_PMo_12_O_40_, ≥99%) and molybdenyl acetylacetonate [Mo(acac)_3_, 99%] were purchased from Sigma-Aldrich. Ethanol (≥99.8%) and deionized (DI) water were used as solvents. All reagents were of analytical grade and used without further purification. In a typical procedure, 0.05 g of phosphomolybdic acid (PMA) and 0.25 g of molybdenyl acetylacetonate [Mo(acac)_3_] were dissolved in a mixed solvent of 31 mL of deionized water and 9 mL of ethanol. The solution was magnetically stirred at room temperature for 20 min to achieve a homogeneous mixture. The resulting solution was then transferred into a Teflon-lined stainless-steel autoclave and heated in an oven at 180 °C for 20 h. After naturally cooling to room temperature, the obtained powder was collected and washed several times with ethanol by centrifugation at 5000 rpm for 7 min to remove any unreacted residues or impurities. The final product was dried at 60 °C overnight prior to further characterization.

### Sensing experiment

The photophysical potential of the synthesized PMO toward the selective detection of metal ions was investigated using UV-visible spectroscopy. In this study, various metal salts belonging to alkali, alkaline earth, and transition metal ions were screened, including Na^+^, Cu^2+^, Ca^2+^, Ba^2+^, Mg^2+^, Al^3+^, Pb^2+^, Ni^2+^, Zn^2+^, and Hg^2+^. For each sensing experiment, 1 mM salt solution was used and mixed with PMO in an equal volume ratio (1 : 1, v/v) and stirred at room temperature for 10 min, after which the absorption spectra were recorded using a UV-visible spectrophotometer. Among the tested metal ions, Pb^2+^ induced a rapid colorimetric response within 5 min, evidenced by the gradual fading of the initial color of the PMO solution, indicating strong interaction between Pb^2+^ and PMO. To find out the limit of detection for Pb^2+^, concentration study experiments were conducted. Keeping the concentration of PMO constant, Pb^2+^ concentration was changed from 10–100 µM. The limit of detection was determined using slope of straight line equation. Selectivity of PMO towards Pb^2+^ was determined using competitive experiments. The change in the absorption intensity of nanoparticles upon addition of competitive metal ions in presence of particular target analyte was recorded. The aim of this study is to determine the selectivity of PMO for Pb^2+^ in presence of other similar metal ions. For this purpose, 1 mM aqueous solutions of each metal ion were prepared and mixed with PMO and Pb^2+^ in an equal volume ratio (1 : 1 : 1, v/v). The resulting mixtures were stirred at room temperature for 10 min prior to recording the absorption spectra using a UV-visible spectrophotometer. The effect of pH was examined using a 100 µM salt solution prepared in phosphate-buffered saline (PBS), with the pH adjusted using HCl and NaOH for acidic and basic conditions, respectively. Temperature-dependent sensing behavior and thermal stability of PMO and the PMO–Pb^2+^ system were evaluated over a temperature range of 20–100 °C, and the corresponding UV-visible absorption spectra were recorded after thermal equilibration at each temperature.

### Characterization

The morphology and microstructure of the synthesized products were examined using high-resolution transmission electron microscopy (HRTEM) and dark-field scanning transmission electron microscopy (STEM) on a Field Electron and Ion Company (FEI) Tecnai G2 F20 S-Twin microscope at an accelerating voltage of 200 kV. Energy-dispersive X-ray (EDX) element mapping tests were also performed on the same instrument to analyze the elemental distribution within the nanostructures. Scanning electron microscopy (SEM) images were obtained using a scanning electron microscope (Hitachi SU8010, Japan) equipped with an energy-dispersive X-ray spectroscope (EDX) to observe the three-dimensional architecture and local elemental composition of the superstructures. The crystalline structure and mesoscale ordering were investigated by X-ray diffraction (XRD) using a Bruker D8 Advance X-ray diffractometer with Cu Kα radiation (*λ* = 1.5418 Å). Fourier-transform infrared (FTIR) spectroscopy was carried out using a PerkinElmer FT-IR spectrophotometer over the range of 4000–400 cm^−1^ with a resolution of 4 cm^−1^ using the KBr pellet method to identify functional groups and chemical bonding. X-ray photoelectron spectroscopy (XPS) was employed to determine the surface chemical composition and oxidation states of the elements using a Thermo Fisher ESCALAB 250Xi spectrometer applying monochromatic Al Kα X-ray sources (1486.6 eV) at 2.0 kV and 20 mA. All binding energies were calibrated using the C 1s peak from adventitious carbon at 284.8 eV. UV-vis absorption spectra for the sensing experiments were recorded using a UV-2600 spectrophotometer (Shimadzu, Kyoto, Japan) over a wavelength range of 200–800 nm.

## Results and discussion

**Fig. 1:**
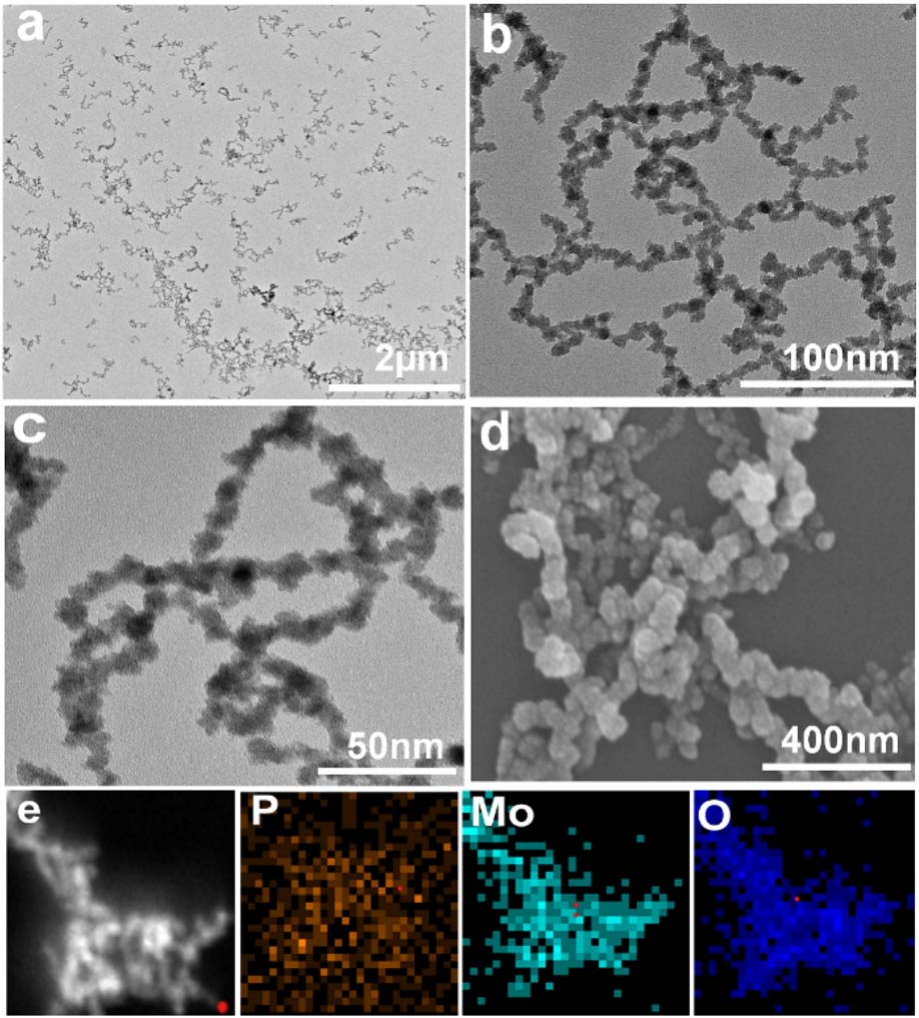
Morphological and elemental characterization of the synthesized PMO nanostructures. (a) Low-magnification TEM image. (b) TEM image at higher magnification. (c) Further magnified TEM image. (d) SEM image. (e) STEM image and corresponding EDX elemental mapping of P, Mo and O.

Low-magnification TEM imaging revealed uniformly distributed dendrimer-like architectures over a large area, indicating consistent morphology and high structural purity (Fig. 1a). Higher-magnification image revealing a regular branched network composed of interconnected bead-like features (Fig. 1b). Further magnified view demonstrating that each bead is constructed from uniformly sized primary nanoparticles, confirming hierarchical self-assembly from nanoparticles to spherical bead-like intermediates and subsequently to morphologically dendrimer like structures (Fig. 1c). SEM image highlighting the three-dimensional dendritic morphology with closely packed beads forming continuous frameworks (Fig. 1d). STEM image and corresponding EDX elemental maps of P, Mo, and O, showing homogeneous elemental distribution throughout the PMO structure, confirming compositional uniformity and phase purity (Fig. 1e).

**Fig. 2:**
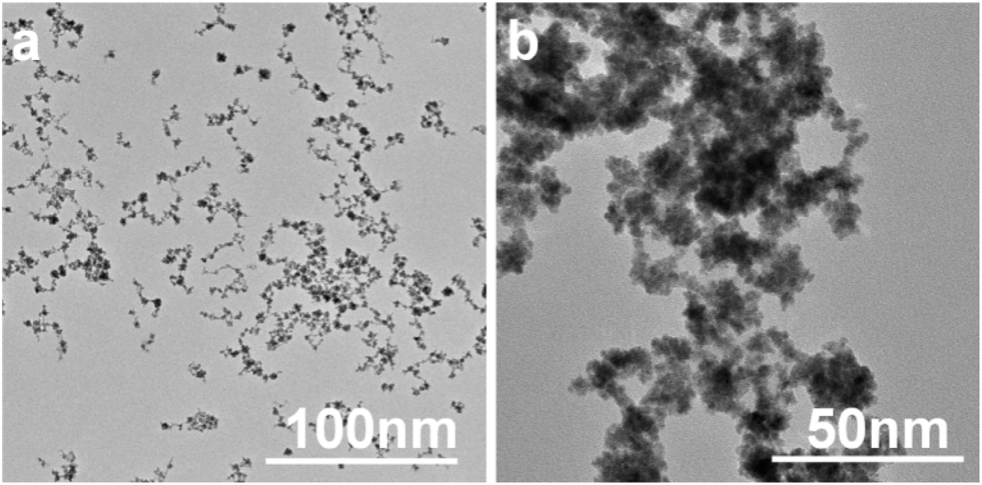
TEM images of the sample synthesized without PMA revealing randomly aggregated particles with poor structural organization (a) low-magnification TEM image (b) higher-magnification TEM image.

Fig. 2 shows low-resolution TEM images of the sample synthesized without PMA, while all other experimental conditions were kept constant. A wide-area view showing randomly distributed nanostructures with irregular shapes and broad size distribution, indicating uncontrolled growth in the absence of a structure-directing agent (Fig. 2a). A higher-magnification image of the same sample revealing agglomerated, disordered assemblies, further confirming the lack of structural regularity without PMA (Fig. 2b).

**Fig. 3:**
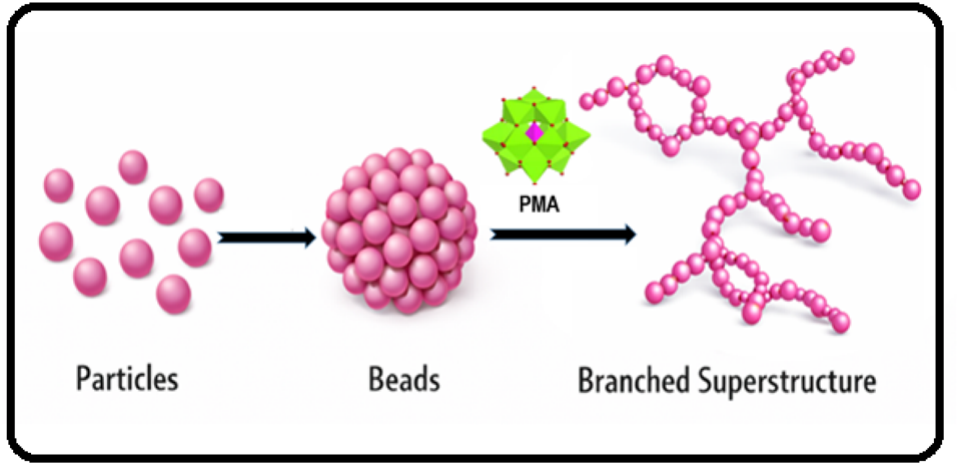
Schematic of the hierarchical assembly (bottom up approach).

Fig. 3 is the schematic of the hierarchical assembly pathway from primary particles to a branched superstructure. The process initiates with monodisperse colloidal particles that first aggregate into spherical bead-like intermediates through specific short-range interactions. These secondary clusters then undergo directional assembly to organize into an open, porous, and dendritic network. Phosphomolybdic acid (PMA) plays a critical structure-directing role in the second stage of the assembly process, guiding the isotropic spherical bead-like intermediates into an anisotropic, three-dimensional branched network. We postulate that the Keggin-type PMA clusters, with their abundant surface oxygen atoms and negative charge, coordinate to the surface of the molybdenum oxide beads. The functional groups of the PMA effectively “activate” specific sites on the bead surface, promoting directional end-to-end and tip-to-side aggregation. This process is analogous to the stepwise, directional growth seen in dendritic macromolecules, where the PMA acts as a multifunctional branching unit. The result is the formation of an open, porous, and hierarchically organized dendrimer-like superstructure, where the original bead-like intermediates become the nodes in a continuous, branched framework.

**Fig. 4:**
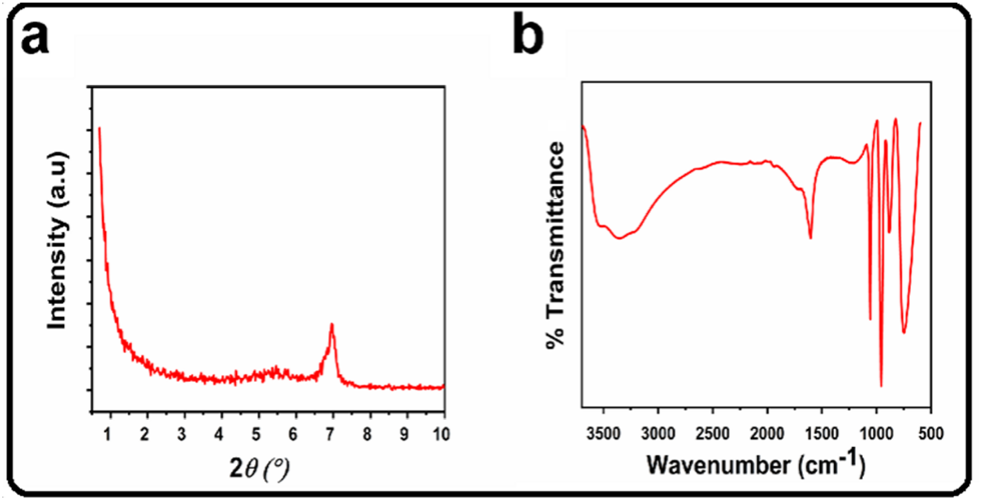
Structural characterization of the PMO material: (a) SAXRD pattern. (b) FTIR spectrum.

Fig. 4 presents complementary structural and chemical characterization of the synthesized mesoporous material, combining small-angle X-ray diffraction (SAXRD) and Fourier-transform infrared (FTIR) spectroscopy. Fig. 4a displays the SAXRD pattern, which confirms the presence of a well-ordered mesoporous structure with long-range periodicity. The pattern exhibits distinct, low-angle diffraction peaks, which are characteristic of highly ordered mesopores. The primary peak at the lowest 2*θ* angle corresponds to the (100) reflection of a hexagonal or similar ordered mesophase, while secondary peaks at higher angles (*e.g.*, (110) and (200) reflections) further validate the symmetry and periodic arrangement of the pores. This ordered porosity is essential for providing a high surface area and uniform active sites, which are critical for the material's intended sensing or adsorption applications. Fig. 4b shows the corresponding FTIR spectrum, providing molecular-level insight into the chemical composition and bonding within the mesoporous framework. The spectrum reveals key absorption bands: a broad band around ∼3500 cm^−1^ corresponds to O–H stretching vibrations, indicative of surface hydroxyl groups or physisorbed water. Sharp bands in the 2850–2950 cm^−1^ range are attributed to C–H stretching vibrations, suggesting the presence of organic templates or functional groups from synthesis. More importantly, the fingerprint region (below 1500 cm^−1^) exhibits prominent absorption features, including a systematic increase in intensity with decreasing wavenumber. These bands are characteristic of metal–oxygen (M–O) stretching and bending vibrations, confirming the formation of the inorganic framework that supports the mesostructure. Specific peaks may also indicate the presence of functional organic groups grafted onto the pore walls, which are often integral to the material's functionality. Together, the SAXRD and FTIR analyses provide comprehensive evidence of the material's dual features: an ordered mesoporous architecture (from SAXRD) and a chemically functionalized framework (from FTIR).

**Fig. 5:**
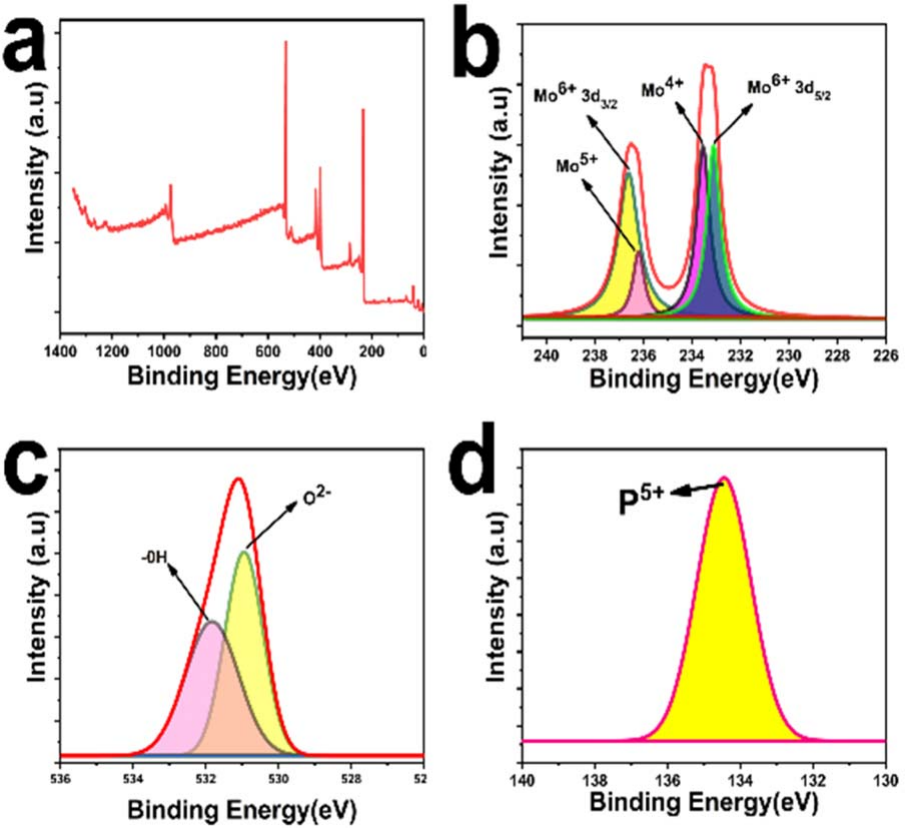
XPS spectra of the PMO: (a) survey scan, (b) Mo 3d, (c) O 1s, and (d) P 2p regions.

The chemical states and surface composition of the PMO superstructure were further elucidated (Fig. 5) by X-ray photoelectron spectroscopy (XPS). The survey spectrum confirmed the presence of all constituent elements (molybdenum, oxygen, and phosphorus) without any detectable impurities (Fig. 5a). The high-resolution Mo 3d core-level spectrum (Fig. 5b) exhibits two prominent peaks at binding energies of 232.2 eV and 235.2 eV, corresponding to the Mo 3d_5/2_ and Mo 3d_3/2_ spin–orbit components, respectively. The spin–orbit splitting of approximately 3.1 eV and the peak positions are characteristic of molybdenum in its highest oxidation state, Mo(vi), confirming the integrity of the molybdenum oxide framework. The O 1s spectrum (Fig. 5c) shows a main peak at 530.1 eV, which is attributed to lattice oxygen (Mo–O) within the inorganic framework. A higher binding energy shoulder, near 531.5 eV, is also observed and can be assigned to surface hydroxyl groups (O–H) and possibly oxygen atoms from the phosphate groups of the PMA. Finally, the P 2p spectrum (Fig. 5d) displays a single, symmetric peak at 133.8 eV, which is consistent with phosphorus in a phosphate (PO_4_^3−^) environment. The presence of this signal provides direct evidence that the phosphomolybdic acid is not merely a free spectator but is successfully integrated into the final PMO superstructure.

**Fig. 6:**
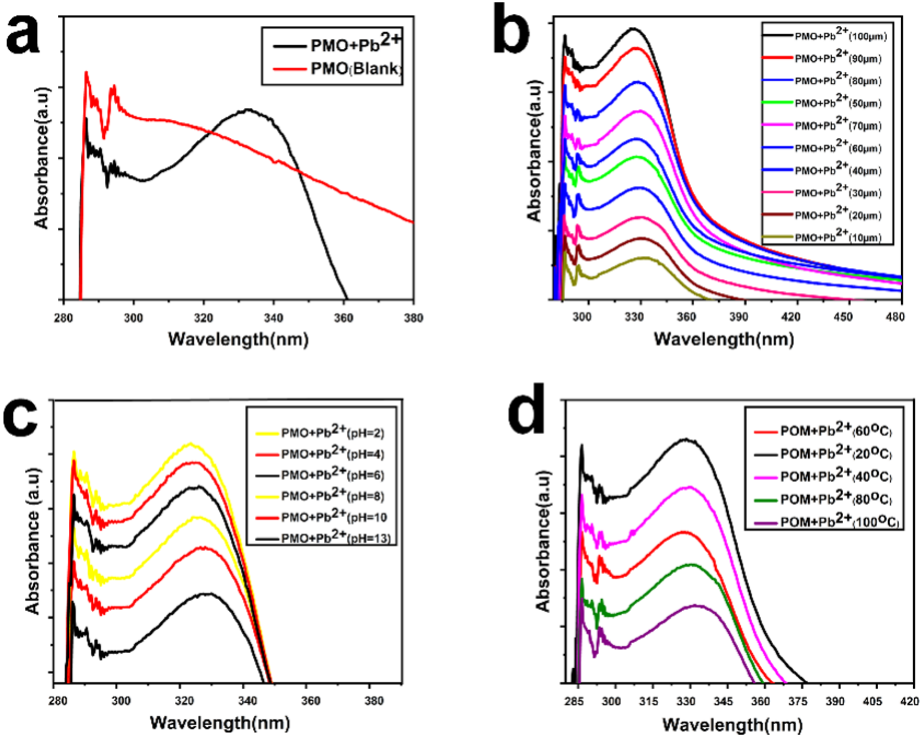
UV-vis absorption spectra demonstrating the sensing performance of PMO toward Pb^2+^ ions. (a) Comparison of PMO in the absence (blank) and presence of Pb^2+^. (b) Absorption response of PMO toward increasing concentrations (10–100 µM). (c) Effect of solution pH^2–13^ on the PMO–Pb^2+^ interaction (d) temperature-dependent UV-vis spectra (20–100 °C) of the PMO–Pb^2+^ system.

The UV-vis absorption behavior of PMO in the presence of Pb^2+^ ions was systematically investigated to evaluate its sensing capability (Fig. 6). As shown in Fig. 6a, the addition of Pb^2+^ to a PMO solution induces a distinct change in the absorbance profile compared to the PMO blank, confirming a specific interaction between the sensor material and the target ion. This foundational response was further characterized by examining its dependence on ion concentration (Fig. 6b), where the absorption intensity of the PMO–Pb^2+^ complex was found to increase systematically with concentration from 10 to 100 µM. The limit of detection was measured by the standard deviation of blank and slop of straight line. This study (10–100 µM) yields a linear calibration curve (*R*^2^ = 0.996) with a limit of detection (LOD) of 0.55 µM (based on 3*σ*/slope). This adherence to the Beer–Lambert law confirms the quantitative nature of the optical signal. The influence of solution pH on the system is pronounced (Fig. 6c), with significant spectral variations observed across a broad pH range.^2–13^ This strong pH dependence suggests that the protonation and deprotonation of the abundant surface hydroxyl groups and bridging oxygen atoms of the molybdenum oxide framework critically modulate Pb^2+^ binding affinity, thereby defining the optimal operational window for detection. The interaction is likely electrostatic or involves chelation with the oxygen-rich framework, where Pb^2+^ binding perturbs the Mo–O charge-transfer bands, leading to the observed spectral changes. Furthermore, temperature-dependent studies (Fig. 6d) demonstrate that the PMO–Pb^2+^ interaction remains stable across a 20–100 °C range, although a gradual decrease in absorbance at elevated temperatures is noted, potentially due to reversible thermal effects on the binding equilibrium. Collectively, these results demonstrate that PMO exhibits a reliable, quantifiable, and environmentally tunable optical response to Pb^2+^, underscoring its strong potential as a robust platform for UV-vis based lead sensing.

**Fig. 7:**
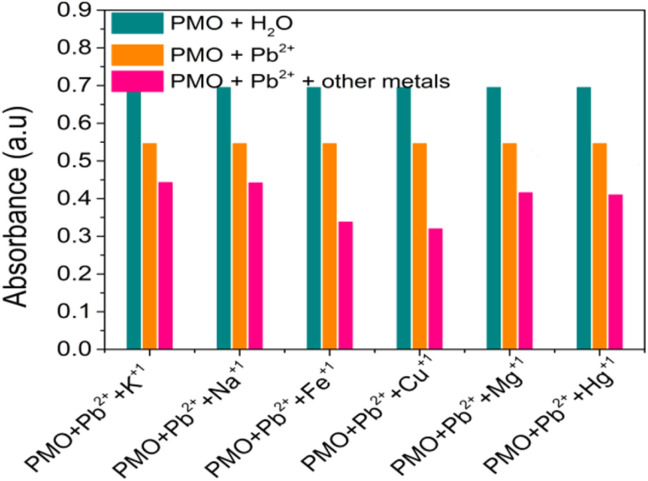
Selectivity of PMO toward Pb^2+^ ions in the presence of various interfering metal ions.

The selectivity of PMO as an optical sensing probe was investigated by comparing its response toward Pb^2+^ and other potentially interfering metal ions under identical conditions. As shown in Fig. 7, PMO exhibits a significantly enhanced absorbance response in the presence of Pb^2+^ compared to the other tested metal ions, indicating a strong and preferential interaction between Pb^2+^ and the functional groups present on the PMO surface. The relatively low response observed for competing ions suggests minimal interference, highlighting the high selectivity of PMO toward Pb^2+^ detection. The enhanced affinity of PMO for Pb^2+^ can be attributed to favorable coordination interactions between Pb^2+^ ions and the oxygen-containing active sites within the mesoporous framework. These results demonstrate the suitability of PMO as a selective sensing platform for the detection of Pb^2+^ in complex aqueous environments.

The detection of Pb^2+^ ions by the PMO material is proposed to occur through a synergistic mechanism involving surface adsorption and redox interaction. The MoOx matrix contains abundant oxygen vacancies and Lewis's acid sites. Pb^2+^ ions, being a soft Lewis acid with high polarizing ability, interact strongly with surface oxygen atoms (especially terminal Mo–O and bridging Mo–O–Mo groups). This leads to electrostatic attraction between Pb^2+^ and negatively charged surface oxo groups and formation of Pb–O–Mo coordination complexes, which partially screen the surface charge. Moreover, PMA is also capable of reversible multi-electron reduction. Pb^2+^ adsorption alters the local electron density around Mo, promoting partial reduction of Mo^6+^ to Mo^5+^. The positively charged Pb^2+^ may stabilize reduced Mo species, shifting the reduction potential of Mo^6+^/Mo^5+^ to less negative values, thereby facilitating spontaneous electron transfer from the support or from adsorbed water/hydroxyl groups.

## Conclusions

We have developed a modular one-step strategy for synthesizing all-inorganic dendrimer-like superstructures through PMA-directed assembly of molybdenum oxide species. The resulting PMO material exhibits hierarchical organization, compositional uniformity, and ordered mesoporosity. PMA plays a crucial role in directing anisotropic aggregation and enabling dendritic growth. Beyond structural novelty, the material demonstrates a reliable and tunable UV-visible response toward Pb^2+^ ions, highlighting its potential in environmental sensing applications. This work establishes a new pathway for constructing morphologically dendrimer-inspired inorganic superstructures without organic templating or multistep synthesis, offering promising opportunities in catalysis, redox chemistry, and chemosensing.

## Author contributions

Fozia Nazir: writing – original draft, methodology, software. Syeda Sundas Musawar: writing – review and editing. Bilal Akram: writing – review and editing, supervision, data curation, investigation, formal analysis, project administration, resources. Ashfaq Ahmad Khan: supervision, investigation.

## Conflicts of interest

There are no conflicts to declare.

## Data Availability

Data can be obtained from corresponding authors upon reasonable request.
